# Chromosomal Microarray Analysis in Fetuses Detected with Isolated Cardiovascular Malformation: A Multicenter Study, Systematic Review of the Literature and Meta-Analysis

**DOI:** 10.3390/diagnostics12061328

**Published:** 2022-05-27

**Authors:** Gioia Mastromoro, Nader Khaleghi Hashemian, Daniele Guadagnolo, Maria Grazia Giuffrida, Barbara Torres, Laura Bernardini, Flavia Ventriglia, Gerardo Piacentini, Antonio Pizzuti

**Affiliations:** 1 Department of Experimental Medicine, Policlinico Umberto I Hospital, Sapienza University of Rome, 00161 Rome, Italy; nader.khaleghihashemian@uniroma1.it (N.K.H.); daniele.guadagnolo@uniroma1.it (D.G.); antonio.pizzuti@uniroma1.it (A.P.); 2 Cytogenetics Unit, Casa Sollievo della Sofferenza Foundation, 71013 San Giovanni Rotondo, Italy; m.giuffrida@css-mendel.it (M.G.G.); b.torres@css-mendel.it (B.T.); l.bernardini@css-mendel.it (L.B.); 3 Department of Pediatrics, Policlinico Umberto I Hospital, Sapienza University of Rome, 00161 Rome, Italy; flavia.ventriglia@uniroma1.it; 4 Santa Maria Goretti Hospital, 04100 Latina, Italy; 5 Fetal and Pediatric Cardiology Unit, “San Giovanni Calibita” Fatebenefratelli Isola Tiberina Hospital, 00186 Rome, Italy; gerardo.piacentini@fbf-isola.it; 6 Neonatology and Neonatal Intensive Care Unit, “San Giovanni Calibita” Fatebenefratelli Isola Tiberina Hospital, 00186 Rome, Italy

**Keywords:** chromosomal microarray analysis, prenatal diagnosis, cardiovascular malformations, genetic counseling, heart disease, fetal malformations, structural anomalies, genetic testing

## Abstract

Cardiovascular malformations (CVM) represent the most common structural anomalies, occurring in 0.7% of live births. The CVM prenatal suspicion should prompt an accurate investigation with fetal echocardiography and the assessment through genetic counseling and testing. In particular, chromosomal microarray analysis (CMA) allows the identification of copy number variations. We performed a systematic review and meta-analysis of the literature, studying the incremental diagnostic yield of CMA in fetal isolated CVM, scoring yields for each category of heart disease, with the aim of guiding genetic counseling and prenatal management. At the same time, we report 59 fetuses with isolated CVM with normal karyotype who underwent CMA. The incremental CMA diagnostic yield in fetuses with isolated CVM was 5.79% (CI 5.54–6.04), with conotruncal malformations showing the higher detection rate (15.93%). The yields for ventricular septal defects and aberrant right subclavian artery were the lowest (2.64% and 0.66%). Other CVM ranged from 4.42% to 6.67%. In the retrospective cohort, the diagnostic yield was consistent with literature data, with an overall CMA diagnostic yield of 3.38%. CMA in the prenatal setting was confirmed as a valuable tool for investigating the causes of fetal cardiovascular malformations.

## 1. Introduction

Cardiovascular anomalies occur in about 0.7% of live births [1,2,3], excluding small muscular ventricular septal defects (VSDs) that are detected in 5–6% of neonates and 95% of these close spontaneously in the first year of life [4]. The prevalence is higher in the prenatal setting because some VSDs identified in utero can close spontaneously before birth. Cardiovascular malformations include a heterogeneous group of anomalies that can present either as an isolated finding or as part of a conspicuous number of chromosomal, genomic or monogenic conditions. Associated structural malformations are not always identified prenatally, with potential repercussions on the clinical outcome. Many of the children diagnosed with congenital heart disease undergo cardiac surgery with favourable results [5]. However, knowing if cardiac malformation is part of a syndromic picture is important for planning the timing of the intervention and for avoiding the most frequent complications in the specific subgroup of patients. According to the literature, in some cases the prognosis depends on the presence of further structural anomalies or associated genetic conditions [6] and knowing the underlying disease can help to predict outcome and complications.

Prenatal suspicion of heart disease during an obstetric ultrasound scan should prompt the investigation of intracardiac and vascular morphology with fetal echocardiography. A careful medical history of the pregnant woman and the whole family can rule out possible underlying causes and guide diagnostic investigations after genetic counseling. Fetal echocardiography is a detailed ultrasound examination focused on the morphology of the heart and cardiovascular system, which can be performed from the late first trimester to term [7], due to fetal factors, maternal or familial diseases or environmental exposure [8].

Genetic counseling allows an overall assessment of the case, based on imaging investigations and on the information acquired by the consultants. Cytogenetics and molecular testing after sample collection can be requested. Conventional karyotyping in some countries is still considered the first-tier test when structural anomalies are detected. It is able to diagnose chromosomal numerical and structural anomalies starting from 5–10 megabases (Mb). Karyotype anomalies are identified in up to 30–40% of fetuses with cardiovascular malformations, and the higher prevalence of chromosomopathies in this population, if compared to postnatal cases, is attributable to the high intrauterine mortality related to these conditions [7]. Chromosomal microarray analysis (CMA) is a tool capable of detecting copy number variations (CNVs) with a 50 Kb resolution. It can be performed in fetuses with structural anomalies after or alongside standard karyotype or as a first-tier test. Fetuses with heart diseases, as well as most structural anomalies, may benefit from this assay, whose incremental diagnostic rate over standard karyotyping ranges from 3.18% (CI 3.11–3.25) to 6.47% (CI 6.23–6.71) in this subgroup of isolated malformations [9]. 

In the literature, most of the series concerning the association between heart malformations and CNVs were postnatally performed. In the prenatal setting, no distinction is usually made between the different diagnostic yields of laboratory techniques with respect to the specific cardiac malformation.

We performed a systematic review and meta-analysis of the literature, studying the incremental yield of CMA in fetal-isolated cardiovascular anomalies and calculating the specific yield based on the category of heart disease, with the aim of guiding genetic counseling after assessment of each subgroup of cardiovascular malformation. At the same time, we compared a series of fetuses with isolated cardiovascular malformation, who showed normal karyotype and underwent CMA, with the literature.

## 2. Materials and Methods

### 2.1. Systematic Review of the Literature

The research was conducted following PRISMA guidelines [10]. We searched the Pubmed database (https://pubmed.ncbi.nlm.nih.gov/), accessed on 25 April 2022 for (“fetus” OR “fetuses” OR “foetus” OR “foetuses” OR “fetal” OR “foetal” OR “prenatal” OR “pre-natal”) AND (“congenital heart disease” OR “congenital heart diseases” OR “congenital heart defect” OR “congenital heart defects” OR “congenital cardiac malformation” OR “congenital cardiac malformations” OR “heart disease” OR “heart diseases” OR “heart defect” OR “heart defects” OR “cardiac malformation” OR “cardiac malformations” OR “Crisscross heart” OR “Dextrocardia” OR “Ebstein Anomaly” OR “Ventricular Septal Defect” OR “Atrial Septal Defect” OR “Aortopulmonary Window” OR “Persistent Truncus Arteriosus” OR “Heterotaxy Syndrome” OR “Heterotaxia” OR “Right Isomerism” OR “Left Isomerism” OR “Atrioventricular Canal” OR “Atrioventricular Septal Defect” OR “Hypoplastic Left Heart Syndrome” OR “Tetralogy of Fallot” OR “Transposition of Great Vessels” OR “Transposition of Great Arteries” OR “Double Outlet Right Ventricle” OR “Double Outlet Left Ventricle” OR “Double Inlet Left Ventricle” OR “Double Inlet Right Ventricle” OR “Tricuspid Atresia” OR “Mitral Atresia” OR “Aortic Stenosis” OR “Aortic Atresia” OR “Pulmonary Stenosis” OR “Pulmonary Atresia”) AND (“molecular cytogenetic” OR “molecular cytogenetics” OR “CMA” OR “chromosomal microarray analysis” OR “comparative genomic hybridization” OR “comparative genomic hybridization” OR “hybridization” OR “hybridization” OR “microarray” OR “microarrays” OR “array” OR “arrays” OR “CGH” OR “array-CGH” OR “array CGH” OR “single nucleotide polymorphism” OR “SNP” OR “SNP-array” OR “microdeletion” OR “microduplication” OR “CNV” OR “CNVs” OR “copy number variant” OR “copy number variants” OR “copy number variation” OR “copy number variations”).

All titles and abstracts were examined. Only papers with full text available in the English language were retained. Papers not describing the prenatal diagnostic application of CMA on invasive samples in fetuses detected with cardiac anomalies were excluded. Case reports were excluded. Cases with postnatal diagnosis, fetuses enrolled for known chromosomal/molecular variants and familiarity/recurrence for genetic disorders, cases enrolled after fetal demise and twin pregnancies were excluded. Papers reporting only cases with associated structural anomalies were excluded. Papers in which these categories were included but could not be separated from eligible cases were secondarily excluded.

Cases from eligible papers were classified into different subgroups based on the type of heart disease. We excluded those conditions suspected in the prenatal period but requiring a postnatal confirmatory diagnosis or dynamic entities that did not meet the definition of fetal structural anomalies, such as aortic coarctation or suspicion of atrial septal defects and imbalanced ventricles [11].

Cases included in this meta-analysis were divided according to the type of heart disease. The incremental yield of CMA over standard karyotyping was assessed for each class of anomaly as “pooled number of cases with a Pathogenic or Likely Pathogenic (P/LP) CNV”/“pooled number of recruited cases”. We excluded cases for which the original paper reported the specific data as statistically non-relevant. We excluded from quantitative analysis and data pooling heart conditions for which less than three papers were available or in which less than thirty fetuses were analyzed. 

Standard deviations and 95% confidence intervals were scored with the = STDEV.S and = CONFIDENCE functions on Microsoft Excel (Office 365).

### 2.2. Retrospective Cohort

We retrospectively collected fetuses detected with isolated cardiac malformations between March 2018 and April 2022. Invasive sampling (chorionic villus of amniotic fluid) was offered in each case. After informed consent, karyotyping and CMA were performed.

We excluded fetuses detected with additional malformations in organs not included in the cardiovascular system and cases in which a monogenic condition was highly suspected based on family history and/or known familial variants. We excluded twin pregnancies because they present additional risks for heart diseases [12]. We noted soft markers and the mode of conception.

Echocardiographic scans were performed by two operators using 3d convex volumetric probe with frequency range of 4–8 MHz of a Samsung Echocardiography Accuvix A30, HS60 and 3d convex volumetric probe with frequency range of 1–8 MHz of a Samsung Medison HS70A Prime, in accordance with the American Society of Echocardiography guidelines for fetal echocardiography [13].

Genomic screening for CNVs was performed on fetal and parental DNA using the Cytoscan HD (Thermo Fisher Scientific, Waltham, MA, USA) or the 180K oligonucleotide-array (Agilent Technologies, Waldbronn, Germany) microarray platform, following the manufacturer’s instructions and using the ChAS (Thermo Fisher Scientific) or Cytogenomics (Agilent Technologies) analysis software, respectively. Both microarray platforms had 75 Kb effective resolution. Rearrangements were confirmed by real-time quantitative PCR. In accordance with the guidelines of the American College of Medical Genetics (ACMG), the detected CNVs were classified as pathogenic (P), probably pathogenic (LP), variants of uncertain significance (VUS), probably benign (LB) or benign (B) [14].

The diagnostic yield was calculated both on the total number of cardiopathic fetuses analyzed and by excluding the pregnancies arising as a result of assisted reproductive technologies, due to the increased risk of cardiac malformations [15].

## 3. Results

### 3.1. Systematic Review of the Literature

**Figure 1:**
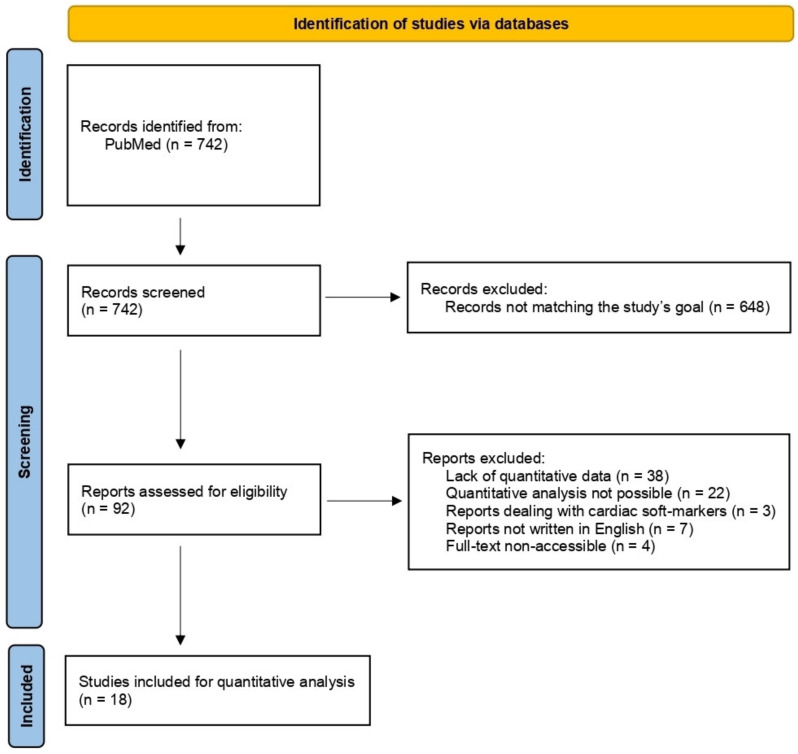
PRISMA flowchart of the systematic review.

The literature search led to the identification of 742 papers. A total of 648 were excluded from the review for not being in line with the objectives of the study. A total of 92 papers were retained and analyzed. A further 74 articles were secondarily excluded from the quantitative analysis. Of these, 38 did not provide quantitative data [6,16,17,18,19,20,21,22,23,24,25,26,27,28,29,30,31,32,33,34,35,36,37,38,39,40,41,42,43,44,45,46,47,48,49,50,51,52], in 22 articles it was not possible to infer a quantitative analysis of incremental yield of CMA over standard karyotyping [53,54,55,56,57,58,59,60,61,62,63,64,65,66,67,68,69,70,71,72,73,74], 3 papers concerned only prenatal cardiac soft-markers and not cardiovascular malformations [75,76,77], 7 articles did not present the full-text in English [78,79,80,81,82,83,84] and the full-text of 4 articles was not accessible [85,86,87,88]. A total of 18 articles were eligible for quantitative analysis [89,90,91,92,93,94,95,96,97,98,99,100,101,102,103,104,105,106] (Figure 1, Table 1).

**Table 1:** Systematic review of incremental diagnostic yield and VUS rate detected by CMA for each subgroup of CVM.

Article (Area)	Test	US Anomaly	Diagnostic Yield		VUSs		Negative Results	
**Fu, 2017** [89] **(China)**	SNP	VSD	4/73	(5.48%)	3/73	(4.11%)	66/73	(90.41%)
**Gindes, 2021** [90] **(Israel)**	CMA	PAVSD + MAPCAs	0/3	(0%)	-	-	3/3	(100%)
**Hureaux, 2019** [91] **(France)**	CGH or SNP	isolated CVM	17/160	(10.63%)	4/160	(2.5%)	139/160	(86.88%)
		Septal Defects	3/38	(7.89%)	1/38	(2.63%)	34/38	(89.47%)
		RVOTD	2/13	(15.38%)	1/13	(7.69%)	10/13	(76.92%)
		LVOTD	2/49	(4.08%)	2/49	(4.08%)	45/49	(91.84%)
		CTD	10/60	(16.67%)	0/60	(0%)	50/60	(83.33%)
**Lazier, 2016** [92] **(Canada)**	CGH	isolated CVM	2/8	(25%)	0/8	(0%)	6/8	(75%)
		HLHS	0/2	(0%)	0/2	(0%)	2/2	(100%)
		IAA	0/2	(0%)	0/2	(0%)	2/2	(100%)
		TOF	1/3	(33.33%)	0/3	(0%)	2/3	(66.67%)
		Hypoplastic RV + PA	1/1	(100%)	0/1	(0%)	0/1	(0%)
**Lee, 2020** [93] **(Korea)**	SNP	D-TGA	2/23	(8.7%)	0/23	(0%)	21/23	(91.30%)
**Lu, 2022** [94] **(China)**	SNP	isolated CVM	18/116	(15.52%)	-	-	98/116	(84.48%)
**Maya, 2017** [95] **(Israel)**	CGH	ARSA	0/36	(0%)	-	-	36/36	(100%)
**Maya, 2020** [96] **(Israel)**	CGH, SNP or hybrid	VSD	6/566	(1.06%)	-	-	560/566	(98.94%)
**Mustafa, 2020** [97] **(USA)**	SNP	isolated CVM	8/118	(6.78%)	-	-	110/118	(93.22%)
**Qiao, 2021** [98] **(China)**	SNP	isolated CVM	18/247	(7.29%)	-	-	229/247	(92.71%)
		VSD	7/92	(7.61%)	-	-	85/92	(92.39%)
		AVSD	1/13	(7.69%)	-	-	12/13	(92.31%)
		DORV	1/17	(5.88%)	-	-	16/17	(94.12%)
		D-TGA	0/13	(0%)	-	-	13/13	(100%)
		IAA B	0/2	(0%)	-	-	2/2	(100%)
		TOF	6/34	(17.65%)	-	-	28/34	(82.35%)
		TA	0/3	(0%)	-	-	3/3	(100%)
		AS	0/4	(0%)	-	-	4/4	(100%)
		HLHS	1/16	(6.25%)	-	-	15/16	(93.75%)
		Ebstein Anomaly	½	(50%)	-	-	1/2	(50%)
		Pulmonary Stenosis	1/16	(6.25%)	-	-	15/16	(93.75%)
		Tricuspid Atresia	0/6	(0%)	-	-	6/6	(100%)
		heterotaxy	0/17	(0%)	-	-	17/17	(100%)
		RAA	0/11	(0%)	-	-	11/11	(100%)
		Cor Triatriatum	0/1	(0%)	-	-	1/1	(100%)
**Sagi-Dain, 2021** [99] **(Israel)**	SNP or hybrid	isolated CVM	27/1365	(1.98%)	-	-	1338/1365	(98.02%)
		VSD	8/623	(1.28%)	-	-	615/623	(98.72%)
		ARSA	3/381	(0.79%)	-	-	378/381	(99.21%)
		RAA	5/136	(3.68%)	-	-	131/136	(96.32%)
		TOF	6/67	(8.96%)	-	-	61/67	(91.04%)
		AVC	1/42	(2.38%)	-	-	41/42	(97.62%)
		TGA	3/30	(10%)	-	-	27/30	(90%)
		Vessel Anomaly	1/27	(3.7%)	-	-	26/27	(96.3%)
		HLHS	0/16	(0%)	-	-	16/16	(100%)
		SIT	0/15	(0%)	-	-	15/15	(100%)
		Interrupted inferior vena cava	0/7	(0%)	-	-	7/7	(100%)
		PLSVC	0/7	(0%)	-	-	7/7	(100%)
		Aortic diameter anomaly	0/6	(0%)	-	-	6/6	(100%)
		DORV	0/5	(0%)	-	-	5/5	(100%)
		TA	0/3	(0%)	-	-	3/3	(100%)
**Shaffer, 2012** [100] **(USA)**	CGH	isolated CVM	4/119	(3.36%)	-	-	115/119	(96.64%)
		HLHS	4/42	(9.52%)	-	-	38/42	(90.48%)
		TOF	0/18	(0%)	-	-	18/18	(100%)
		VSD	0/38	(0%)	-	-	38/38	(100%)
		Dextrocardia/SIT	0/21	(0%)	-	-	21/21	(100%)
**Song, 2019** [101] **(China)**	CMA	isolated CVM	17/138	(12.32%)	10/138	(7.25%)	111/138	(80.43%)
		VSD	8/82	(9.76%)	5/82	(6.1%)	69/82	(84.15%)
		VSD + Aortic Abnormality	3/8	(37.5%)	1/8	(12.5%)	4/8	(50%)
		VSD + Pulmonary Abnormality	1/5	(20%)	0/5	(0%)	4/5	(80%)
		DORV	1/1	(100%)	0/1	(0%)	0/1	(0%)
		VSD, VR	0/1	(0%)	1/1	(100%)	0/1	(0%)
		TOF	1/10	(10%)	0/10	(0%)	9/10	(90%)
		Single Ventricle	2/5	(40%)	0/5	(0%)	3/5	(60%)
		VR	1/21	(4.76%)	1/21	(4.76%)	19/21	(90.48%)
		AS, IAA	0/5	(0%)	2/5	(40%)	3/5	(60%)
**Svirsky, 2019** [102] **(Israel)**	SNP	muscular VSD	0/29	(0%)	-	-	29/29	(100%)
**Turan, 2018** [103] **(USA)**	SNP	isolated CVM	16/86	(18.6%)	-	-	70/86	(81.4%)
		LVOTD	3/22	(13.64%)	-	-	19/22	(86.36%)
		CTD	5/34	(14.71%)	-	-	29/34	(85.29%)
		AVSD	0/2	(0%)	-	-	2/2	(100%)
		VSD	2/7	(28.57%)	-	-	5/7	(71.43%)
		RSD	3/13	(23.08%)	-	-	10/13	(76.92%)
		RAA	2/6	(33.33%)	-	-	4/6	(66.67%)
		ASD + PLSVC	½	(50%)	-	-	1/2	(50%)
**Wang, 2018** [104] **(China)**	SNP	isolated CVM	27/359	(7.52%)	-	-	332/359	(92.48%)
		VSD	6/169	(3.55%)	-	-	163/169	(96.45%)
		TA	3/6	(50%)	-	-	3/6	(50%)
		IAA B	3/11	(27.27%)	-	-	8/11	(72.72%)
		D-TGA	0/11	(0%)	-	-	11/11	(100%)
		DORV	2/11	(18.18%)	-	-	9/11	(81.81%)
		TOF	8/63	(12.7%)	-	-	55/63	(87.30%)
		HLHS	1/16	(6.25%)	-	-	15/16	(93.75%)
		AS	1/3	(33.33%)	-	-	2/3	(66.67%)
		AS + Pulmonary Stenosis	0/2	(0%)	-	-	2/2	(100%)
		Pulmonary Stenosis	1/16	(6.25%)	-	-	15/16	(93.75%)
		Pulmonary Atresia	0/9	(0%)	-	-	9/9	(100%)
		Tricuspid Atresia	0/3	(0%)	-	-	3/3	(100%)
		AVSD	0/4	(0%)	-	-	4/4	(100%)
		Heterotaxy	2/19	(10.53%)	-	-	17/19	(89.47%)
		Single Ventricle	0/1	(0%)	-	-	1/1	(100%)
		L-TGA	0/1	(0%)	-	-	1/1	(100%)
		RAA	0/9	(0%)	-	-	9/9	(100%)
		PLSVC	0/2	(0%)	-	-	2/2	(100%)
		DAA	0/2	(0%)	-	-	2/2	(100%)
		Aortopulmonary window	0/1	(0%)	-	-	1/1	(100%)
**Wu, 2020** [105] **(China)**	CMA	ARSA	0/35	(0%)	0/35	(0%)	35/35	(100%)
		RAA	1/19	(5.26%)	0/19	(0%)	18/19	(94.74%)
**Zhu, 2016** [106] **(China)**	CMA	isolated CVM	6/58	(10.34%)	2/58	(3.45%)	50/58	(86.21%)
		CTD	3/19	(15.79%)	1/19	(5.26%)	15/19	(78.95%)
		AVSD	0/3	(0%)	0/3	(0%)	3/3	(100%)
		LVOTD	0/4	(0%)	0/4	(0%)	4/4	(100%)
		RVOTD	¼	(25%)	1/4	(25%)	2/4	(50%)
		VSD	2/28	(7.14%)	0/28	(0%)	26/28	(92.86%)

**ARSA**: aberrant right subclavian artery; **AS**: aortic stenosis; **AVC**: atrioventricular canal; **AVSD**: atrioventricular septal defect; **CGH**: comparative genomic hybridization; **CMA**: chromosomal microarray analysis; **CTD**: conotruncal defect; **CVM**: cardiovascular malformation; **DAA**: double aortic arch; **D-TGA**: dextro-transposition of the great arteries; **DORV**: Double outlet right ventricle; **HLHS**: hypoplastic left heart syndrome; **IAA**: interrupted aortic arch; **LVOTD**: left ventricular outflow tract defect; **L-TGA**: levo-transposition of the great arteries; **MAPCAs**: major aortopulmonary collateral arteries; **PA**: pulmonary atresia; **PAVSD**: pulmonary atresia with ventricular septal defect; **PLSVC**: persistent left superior vena cava; **RAA**: right aortic arch; **RV**: right ventricle; **RVOTD**: right ventricular outflow tract defect; **RSD**: right sided defect; **SIT**: situs inversus; **SNP**: single-nucleotide polymorphism; **TA**: tricuspid atresia; **TOF**: tetralogy of Fallot; **TGA**: transposition of the great arteries; **US**: ultrasound; **VR**: vascular ring; **VSD**: ventricular septal defect; **VUS**: variants of uncertain significance.

We scored the incremental diagnostic yield of CMA over standard karyotyping for pooled isolated cardiovascular malformations (from papers enrolling all fetuses presenting any cardiovascular malformation) and for eight different individual cardiovascular malformations. Data concerning VUS rates did not meet the criteria for a meta-analysis. The incremental diagnostic yield in fetuses with isolated cardiovascular malformations was 5.79% (5.54–6.04), with a 95% confidence interval (Table 2).

**Table 2:** Meta-analysis of each subgroup of CVM.

CVM Type	References	Diagnostic Yield (95% CI)
**Any CVM**	[91,92,94,97,98,99,100,101,103,104,106]	5.79% (5.54–6.04)
**CTD**	[91,103,106]	15.93% (15.75–16.11)
**TOF**	[92,98,99,100,101,104]	11.28% (9.7–12.86)
**LVOTD**	[91,103,106]	6.67% (5.51–7.83)
**HLHS**	[92,98,99,100,104]	6.52% (5.64–7.4)
**D-TGA**	[93,98,99,104]	6.49% (5.26–7.72)
**RAA**	[98,99,103,104,105]	4.42% (2.36–6.48)
**VSD**	[89,91,96,98,99,100,101,102,103,104,106]	2.64% (2.26–3.02)
**ARSA**	[95,99,105]	0.66% (0.62–0.7)

**ARSA**: aberrant right subclavian artery; **CTD**: conotruncal defect; **CVM**: cardiovascular malformation; **D-TGA**: dextro-transposition of the great arteries; **HLHS**: hypoplastic left heart syndrome; **LVOTD**: left ventricular outflow tract defect; **RAA**: right aortic arch; **TOF**: tetralogy of Fallot; **VSD**: ventricular septal defect.

### 3.2. Retrospective Cohort

We selected 59 fetuses (33 males and 26 females), detected with isolated cardiovascular malformations and normal karyotype, who underwent CMA (Table 3). Five fetuses were conceived through assisted reproductive technologies, in particular, three of the couples underwent fertilization in vitro and embryo transfer (FIVET) and two women underwent intracytoplasmic sperm injection (ICSI). Seven fetuses were detected with soft markers (four with choroid plexus cysts, one with hyperechogenic bowel, one with increased nuchal translucency, and one with short femur and borderline ventriculomegaly) and polyhydramnios was also detected in one of these.

Forty-eight fetuses presented with intracardiac malformations, yielding the following CMA results: eighteen ventricular septal defects (VSD; CMA diagnostic yield 0/18; VUS 3/18), six tetralogy of Fallot (TOF; CMA diagnostic yield 1/6; VUS 0/6), two tricuspid anomalies (CMA diagnostic yield 0/2; VUS 0/2), two truncus arteriosus (TA; CMA diagnostic yield 0/2; VUS 0/2), two hypoplastic left heart syndrome (HLHS; CMA diagnostic yield 0/2; VUS 0/2), two pulmonary atresia with intact ventricular septum (PA-IVS; CMA diagnostic yield 0/2; VUS 0/2), two pulmonary atresia with ventricular septal defect (PA-VSD; CMA diagnostic yield 1/2; VUS 0/2), two aortic valve anomalies (CMA diagnostic yield 0/2; VUS 0/2), one pulmonary stenosis (PS; CMA diagnostic yield 0/1; VUS 0/1), one dysplastic mitral valve (CMA diagnostic yield 0/1; VUS 0/1), one L-looped transposition of great arteries (L-TGA; CMA diagnostic yield 0/1; VUS 0/1), one D-looped transposition of great arteries (D-TGA; CMA diagnostic yield 0/1; VUS 0/1), one atrioventricular canal (AVC; CMA diagnostic yield 0/1; VUS 0/1), one partial atrioventricular canal (pAVC; CMA diagnostic yield 0/1; VUS 0/1), one univentricular heart (CMA diagnostic yield 0/1; VUS 0/1), one left interatrial membrane (CMA diagnostic yield 0/1; VUS 1/1), one TOF with absent pulmonary valve (D-TGA; CMA diagnostic yield 0/1; VUS 0/1) [107], one endocardial fibroelastosis and aortic stenosis (CMA diagnostic yield 0/1; VUS 0/1), one AVC, TGA, PS (CMA diagnostic yield 0/1; VUS 0/1), one D-TGA and tricuspid atresia (CMA diagnostic yield 0/1; VUS 0/1) and one L-TGA, VSD, PS (CMA diagnostic yield 0/1; VUS 0/1).

Twenty-two fetuses were detected with extracardiac cardiovascular malformations, yielding a VUS in one case of right aortic arch (RAA). The cohort encompassed six cases of RAA, four hypoplastic aortic arch, four RAA with aberrant left subclavian artery (ALSA), one RAA with discontinuous pulmonary arteries, one double aortic arch (DAA), one aberrant right subclavian artery (ARSA), one aortic dextroposition, one hypoplastic left pulmonary artery (LPA) with persistent left superior vena cava (PLSVC), one ARSA with PLSVC, one hypoplastic aortic arch with PLSVC and one narrowing of aortic isthmus with PLSVC.

Overall the incremental diagnostic yield for CMA was 2/59 (3.38%), with 6/59 (10.17%) VUSs and 51 (86.44%) negative results. Excluding fetuses conceived with assisted reproductive technologies, which can increase the rate of cardiac malformations, the diagnostic yield was 2/54 (3.70%), with 6/54 (11.11%) VUSs and 46 (85.19%) negative results.

In particular, one likely pathogenic and one pathogenic result were reported. One male fetus, presenting with PA-VSD and whose medical records report increased nuchal translucency in the first trimester examination, was diagnosed with 22q11.2 deletion syndrome. One female fetus with TOF was detected with a 10.7 Mb 21q21.1q21.3 deletion.

## 4. Discussion

The association between cardiac malformations and genetic conditions is widely known. Cytogenetics and molecular investigations are therefore fundamental to guaranteeing the couple an informed choice about the ongoing pregnancy, to formulate the recurrence risks and to guide the most appropriate obstetric management and genetic counseling.

Every genetic investigation must be preceded and followed by non-directive genetic counseling with the aim of educating consultants about the possible conditions related to the malformation and the available testing, providing the basis for thoughtful decision making, according to psychological, socio-economic, cultural and religious backgrounds [9].

CMA allows the detection of small rearrangements that can underlie several structural anomalies [108,109,110,111,112]. This technique has largely replaced conventional karyotyping, becoming the first-tier genetic investigation after the detection of fetal structural anomalies in several countries, even if the most used approach (array-comparative genomic hybridization: a-CGH) is not able to identify balanced chromosomal aberrations, triploidies and mosaicisms below 30%. Some authors suggest replacing standard karyotype with CMA, but also adding a rapid method for detection of aneuploidies and triploidies (e.g., quantitative fluorescent polymerase chain reaction) [113]. In our opinion, it is important to include karyotype examination in any case, due to the possibility of low-rate mosaicism and the importance of chromosomal structure analysis for recurrence risks. However, the abnormal result of a rapid method for detection of aneuploidies and triploidies can avoid the use of CMA in fetuses with these conditions, reducing both turnaround time and costs.

CMA presents a higher resolution and a faster turnaround time, not requiring cell culturing [114], and it is based on different platforms: bacterial artificial chromosome, oligonucleotide and single nucleotide polymorphism-array (SNP-array) [115,116]. Using the first two array platforms, DNA of patient and control samples are marked through different fluorochromes and hybridized to complementary probes on a chip. The colours’ intensity are compared, revealing the presence of CNVs [22]. a-CGH and SNP-array analysis show similar sensitivities in detection of CNVs [117], but SNP-array, using probes with alternative alleles (A and B) of given polymorphic loci, allows the detection of triploidies, lower-level mosaics compared to a-CGH and regions of homozygosity, which can suggest consanguinity or uniparental disomy. CMA is a genome-wide quantitative analysis. This means that it can result in the detection of incidental findings, such as variants in a susceptibility locus for neurodevelopmental disorder, regions encompassing genes associated with diseases with incomplete penetrance and predisposition to late-onset diseases or to variants of uncertain significance (VUSs), such as rearrangements involving candidate genes for disease association [22]. During pretest genetic counseling, the couple should be informed about the possibility of obtaining these results. It is known that unexpected findings can cause a negative psychological impact (anxiety, improper pregnancy management) and clarity on the possible results can encourage the couple to adopt a more rational attitude [118].

### 4.1. CMA in the Literature and in the Present Cohort

In a recent meta-analysis, we calculated the P/LP CMA rate in pregnancies without indications for chromosomal analysis and in advanced maternal age cohorts, which amount, respectively, to 0.79% and 0.84% [9].

In the same work we extrapolated the CMA diagnostic rate of fetuses with cardiovascular malformations from cohorts of fetuses enrolled for any structural anomaly and from papers enrolling fetuses specifically for cardiac defects, yielding different percentages: 3.18% and 6.47%. In particular, in the second group cardiovascular anomalies had the highest detection rate, 6.47% if isolated, compared to the other malformations. This difference prompted us to investigate the detection rates of the subgroups of heart disease. Concerning the detection of VUSs, they represented 2.49% in the first group and 4.74% in the second group.

In the present systematic review and meta-analysis of the literature, the overall detection rate of CMA for pooled isolated cardiovascular malformation was 5.79%, representing a high yield, which differs significantly from the rate obtained from papers enrolling all fetuses for any isolated structural anomaly (3.66–5.64%) [9], while it approaches the percentage obtained from fetuses specifically enrolled in heart disease cohorts from the previous meta-analysis. The reason for this discrepancy can be identified in the different inclusion criteria and in the different approaches that the clinician may have. 

Among the subgroups of heart diseases, the one that showed the highest detection rate was TOF (11.28%). It is one of the most common heart diseases in 22q11.2 deletion syndrome, along with other cardiovascular (especially conotruncal) malformations, such as PA-VSD, TA and interrupted aortic arch [119]. The strong association with this genetic condition could explain the higher diagnostic yield of CMA in this heart disease. Since the diagnostic rate of TOF is so high, it is not surprising that it also appears high in overall conotruncal heart disease, amounting to 15.93%.

The diagnostic yield of VSDs was, as expected, lower than most of the other heart diseases analyzed.

The yield of ARSA was 0.66%, resulting even lower than the sample of fetuses without structural anomalies. It is a frequent vascular anomaly in the population, often misdiagnosed and identified as an incidental finding following investigations carried out for another cause. Although the diagnostic yield is so low, it is important to remember that in the prenatal setting it is not always possible to identify all the morphological anomalies. Due to these limitations that it is still advisable to undergo diagnostic investigations since an anomaly of this type is identified.

The other subgroups of heart disease (RAA, D-TGA, HLHS and left ventricle outflow tract defect) present a detection rate ranging from 4.42% to 6.67%, consistent with the average yield for cardiopathies.

In the retrospective cohort we enrolled, the diagnostic yield was consistent with the data extrapolated from the literature. Overall, the CMA diagnostic yield amounts to 3.38%, with 10.17% VUSs. Excluding fetuses conceived with assisted reproductive technologies, which can increase the rate of cardiac malformations, the diagnostic yield was 3.70%, with 11.11% VUSs.

### 4.2. CMA Compared to Other Techniques

We recently performed a review of the literature scoring the incremental detection rate of prenatal exome sequencing (ES) over CMA [120] and a meta-analysis focusing on the comparison of the available molecular techniques in the prenatal setting [9]. In the first review [120] we analyzed the cohorts of fetuses enrolled for ultrasound anomalies (regardless of the affected organ). The incremental diagnostic yield of prenatal exome sequencing of the included papers ranges from 9% to 47% (average 28%), with a higher rate for fetuses showing multiple malformations. We also analyzed the subgroups of fetuses selected for specific anomalies. In this case, the diagnostic yield ranged from 6% to 92% (average 32%). In particular, the detection rate of heart malformations was 11%, and it seemed to represent the most solid prediction because it was consistent among vast cohorts [98,121,122,123,124]. In the second review and meta-analysis [9] we analyzed cohorts of fetuses enrolled isolated anomalies. In this case, the pooled diagnostic rate for ES was 14.77% (13.23–16.32%) in the group of any cardiovascular anomaly. The rate for isolated or non-isolated cardiovascular anomalies in papers focusing on this subtype of malformation was 11.02% (10.65–11.39%). The high incremental rate of ES is promising, but the possible clinical impact of these diagnoses is still unclear, and the management of molecular VUS, which occur at a high rate, still poses limits to its widespread application.

### 4.3. Proposed Diagnostic Workflow in Fetal Cardiovascular Malformations

In order to avoid inaccurate or fragmentary information, the achievement of a well-defined morphological analysis plays a pivotal role in pretest genetic counseling.

**Figure 2:**
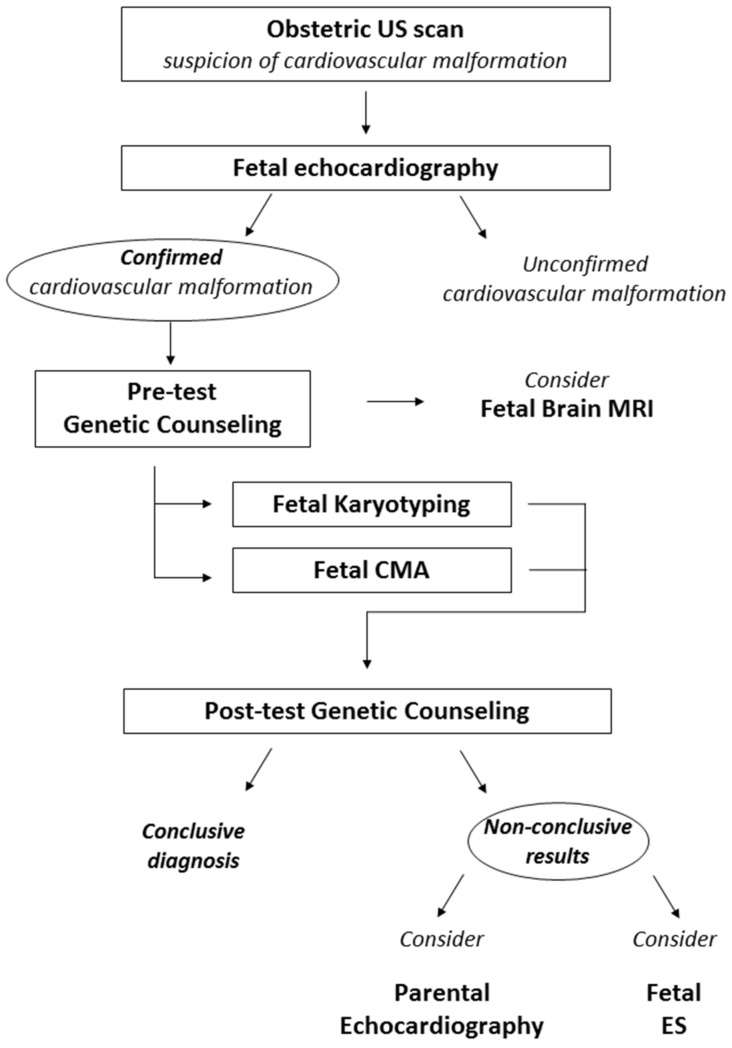
Proposed diagnostic workflow in fetal CVM. The suspicion of a cardiovascular anomaly should always be confirmed with fetal echocardiography. Fetal Karyotyping and CMA should be proposed as first-tier genetic investigation. We also suggest considering further investigations which lack the bulk of evidence for systematic application but can prove to be useful in selected cases. Imaging assessments to be considered include a fetal brain MRI and the study of parental heart morphology with US examination. Fetal ES should be considered in cases with high suspicion of an underlying monogenic cause after non-conclusive karyotyping and CMA. US: ultrasound; MRI: magnetic resonance imaging; CMA: chromosomal microarray analysis; ES: exome sequencing.

Ideally the first genetic counseling should be performed immediately after the suspect of the structural anomaly. If such tempestivity is not possible, the highest priority is a fetal echocardiography, in order to validate or disconfirm the suspicion. Furthermore, with the proper definition of the anatomy of the fetal cardiovascular system, genetic counseling can be targeted on the specific heart anomaly and on the possibly related syndromic conditions. The detection of a fetal cardiovascular anomaly should prompt the proposal of further examination, either of the fetus or the parents. These investigations can include morphological evaluation or genetic testing (Figure 2).

During the pretest genetic counseling, familial history of the couple is gathered and represented graphically as a pedigree. All exams already performed are collected. After the detection of fetal cardiovascular anomalies, standard and molecular karyotyping (CMA) should be requested. These approaches provide complementary information and should not be mutually exclusive [9].

Brain magnetic resonance imaging (MRI) of the fetus should be considered. In fact, central nervous system anomalies often coexist with cardiac malformations, occurring in several syndromes. This can be useful to better define the clinical picture that the fetus will present after birth, if abnormalities of karyotype or CMA are found, or it can guide the analysis of specific monogenic disorders. Moreover, recent studies have confirmed that brain and cerebellar involvement secondary to heart disease can occur, but the clinical consequences of such findings are still under investigation [125,126,127,128].

If both karyotype and CMA yield inconclusive results, the echocardiography of parents and siblings can be taken into consideration. In our experience, it is not uncommon to find slight morphological anomalies or anatomical variants of the main structures of the cardiovascular system, which may be ascribed to the same spectrum of the fetal heart disease, guiding further molecular investigations. Data concerning the cost-effectiveness of this approach and the potential benefit of a systematic application are still lacking.

Post-test genetic counseling should be performed after the results of cytogenetic testing. During this counseling, all investigations carried out should be summarized and discussed.

Although Mendelian inheritance patterns are usually not observed, in the last decade an increasing number of families with cardiac malformations due to monogenic conditions has been described [16], and fetal exome sequencing shows promising diagnostic yields, despite a high VUS burden. For this reason, if there was a suspicion of a monogenic condition, it would be advisable to perform fetal exome sequencing, whose incremental detection rate from CMA amounts 11–14% in fetuses with cardiovascular anomalies [9,120].

## 5. Conclusions

CMA in the prenatal setting represents a valuable tool for investigating the causes of fetal cardiovascular malformations. The overall diagnostic incremental yield of CMA in pooled cardiovascular anomalies accounts for 5.79% and is therefore higher than the average for structural anomalies, confirming the importance of this tool. Most of the heart diseases analyzed (RAA, D-TGA, HLHS and left ventricle outflow tract defect) presented a detection rate ranging from 4.42% to 6.67%, not deviating excessively from the overall rate for cardiopathies. The heart disease that showed the highest detection rate was TOF (11.28%), probably due to the association with 22q11.2 deletion syndrome. Since the diagnostic rate of TOF is so high, it is not surprising that it also appears high in overall conotruncal heart disease, amounting to 15.93%. Despite the yield of ARSA amounts of 0.66%, it is important to perform diagnostic investigations, because not always it is possible to identify all the morphological anomalies in the prenatal setting. In the retrospective cohort, diagnostic yield was consistent with the data extrapolated from the literature. The prenatal management of CVMs is challenging for all professionals involved. Providing couples with knowledge of the specific risks for each malformation can be extremely valuable in a tailored genetic counseling. 

## Figures and Tables

**Table 3:** Retrospective cohort of fetuses with isolated CVM.

Case	Sex	Intracardiac Anomaly	Extracardiac Anomaly	CMA	Soft Markers or Fluid Anomaly	Mode of Conception
1	M	PS	---	N	---	S
2	F	VSD	---	N	---	S
3	M	L-TGA	---	N	---	S
**4**	**F**	**TOF**	---	**del21q21.1q21.3 (10.7 Mb)**	---	**S**
5	M	muscular VSD	---	N	---	S
6	M	VSD	---	N	---	S
7	M	TA type 2	---	N	choroid plexus cyst	S
8	F	AVC, TGA, PS	---	N	---	S
9	F	Tricuspid Atresia	---	N	---	S
10	F	HLHS	---	N	---	S
11	F	Univentricular Heart	---	N	---	S
12	F	PA-IVS	---	N	---	S
13	F	left interatrial membrane	---	21q22.3(47663694-47931362) × 3 mat	choroid plexus cyst	S
14	M	---	RAA	N	---	S
15	F	---	RAA	1q42.3(235810562-236471639) × 3 pat 4q13.2q13.3(70405132-70917149) × 1 pat	---	S
16	F	aortic valve stenosis	---	N	---	S
17	F	---	RAA	N	---	S
18	M	muscular VSD	---	1q25.1(173390879-173855070) × 3 pat	choroid plexus cyst	S
19	M	D-TGA	---	N	---	S
20	M	---	RAA	N	---	S
21	M	TOF	RAA, disc PAs	N	choroid plexus cyst	S
22	F	VSD	---	9q34.3(139362970-139432609) × 3 pat 17p12(14700924-15257475) × 3 pat	hyperechogenic bowel	S
23	F	---	RAA	N	---	S
24	F	TOF	---	N	---	ICSI
**25**	**M**	**PA-VSD**	---	**22q11.2(18651614-21464119)x1**	**increased nuchal translucency**	**S**
26	F	dysplastic mitral valve	---	N	---	S
27	M	TA type 1°	---	N	---	S
28	F	hypoplastic aortic valve	hypoplastic aorta	N	---	S
29	M	VSD	hypoplastic LPA, PLSVC	Xp11.23(590 kb) × 3 mat	---	S
30	M	subaortic VSD, overriding aorta	ARSA, PLSVC (absent right SVC)	N	---	S
31	M	TOF	---	N	---	S
32	M	PA-IVS	---	N	---	S
33	M	---	DAA	N	---	S
34	M	VSD	---	N	short femur length, borderline ventriculomegaly	S
35	F	endocardial fibroelastosis, aortic stenosis	---	N	---	S
36	M	PA-VSD	RAA	N	---	S
37	M	TOF	---	N	---	ICSI
38	M	VSD	hypoplastic aortic arch, PLSVC	N	polyhydramnios	S
39	M	subaortic VSD	---	4q31.22q31.23(148380556-148787969) × 3 pat	---	S
40	M	---	ARSA	N	---	S
41	F	D-TGA, Tricuspid atresia	hypoplastic aortic arch	N	---	FIVET
42	F	pAVC	---	N	---	S
43	M	tricuspid dysplasia	---	N	---	S
44	F	muscular VSD	RAA, ALSA	N	---	FIVET
45	M	L-TGA, VSD, PS	hypoplastic aortic arch	N	---	S
46	F	AVC	hypoplastic aortic arch	N	---	FIVET
47	F	muscular VSD	---	N	---	S
48	M	---	aortic dextroposition, aortic kinking	N	---	S
49	M	TOF	---	N	---	S
50	M	---	vascular ring (RAA and LDA), ALSA	N	---	S
51	M	apical muscular VSD	---	N	---	S
52	F	---	RAA, ALSA, Kommerell diverticulum	N	---	S
53	F	subaortic and muscular VSDs	---	N	---	S
54	M	muscular VSD	---	N	---	S
55	F	subaortic VSD	---	N	---	S
56	F	---	RAA, ALSA, Kommerell diverticulum	N	---	S
57	M	TOF, APV	---	N	---	S
58	M	VSD	narrowing of aortic isthmus, PLSVC	N	---	S
59	M	HLHS	---	N	---	S

**ARSA**: aberrant right subclavian artery; **AS**: aortic stenosis; **AVC**: atrioventricular canal; **AVSD**: atrioventricular septal defect; **CMA**: chromosomal microarray analysis; **CVM**: cardiovascular malformation; **DAA**: double aortic arch; **D-TGA**: dextro-transposition of the great arteries; **DORV**: Double outlet right ventricle; **FIVET**: fertilization in vitro embryo transfer; **HLHS**: hypoplastic left heart syndrome; **IAA**: interrupted aortic arch; **ICSI**: intracitoplasmic sperm injection; **L-TGA**: levo-transposition of the great arteries; **PA**: pulmonary atresia; **PAVSD**: pulmonary atresia with ventricular septal defect; **PLSVC**: persistent left superior vena cava; **PS**: pulmonary stenosis; **RAA**: right aortic arch; **RV**: right ventricle; **RVOTD**: right ventricular outflow tract defect; **RSD**: right sided defect; **TA**: tricuspid atresia; **TOF**: tetralogy of Fallot; **TGA**: transposition of the great arteries; **US**: ultrasound; **VR**: vascular ring; **VSD**: ventricular septal defect; **VUS**: variants of uncertain significance.

## Data Availability

The data presented in this study are available in the articles cited in the References section.
